# Regulation of Human Lung Adenocarcinoma Cell Proliferation by LncRNA AFAP-AS1 Through the miR-508/ZWINT Axis

**DOI:** 10.3390/ijms26136532

**Published:** 2025-07-07

**Authors:** Sultan F. Kadasah, Abdulaziz M. S. Alqahtani

**Affiliations:** Department of Biology, Faculty of Science, University of Bisha, P.O. Box 551, Bisha 61922, Saudi Arabia

**Keywords:** lung adenocarcinoma, AFAP1-AS1, invasion, miR-508-3p, ZWINT, proliferation

## Abstract

Lung adenocarcinoma is a prevalent, aggressive cancer with a poor prognosis due to early metastasis and resistance to treatment. LncRNA AFAP1-AS1 has been shown to be associated with the development of multiple carcinomas. This study investigates the functional role of AFAP1-AS1 in lung adenocarcinoma cell proliferation via miR-508-3p and ZWINT. Human lung adenocarcinoma A549 cells were transfected with siRNA constructs against AFAP1-AS1 (si-AFAP1-AS1) to silence its expression. Cell proliferation was evaluated via CCK-8 and colony-forming assays. Apoptosis was assessed using AO/EB staining, and invasion was determined via Transwell assay. The interaction between AFAP1-AS1, miR-508-3p, and ZWINT was confirmed via dual luciferase reporter assay and qRT-PCR analysis. Data were analysed using appropriate statistical tests. AFAP1-AS1 was significantly upregulated in lung adenocarcinoma cells compared to normal BEAS-2B cells. Silencing of AFAP1-AS1 resulted in a marked reduction in A549 cell proliferation and colony development, as observed in CCK-8 and colony formation assays. The AO/EB assay showed a significant increase in apoptosis (30 ± 4.4%) in si-AFAP1-AS1 transfected cells compared to control si-NC (3 ± 1.2%). In addition, knockdown of AFAP1-AS1 led to an upsurge of pro-apoptotic Bax and decline of anti-apoptotic Bcl-2 expression. The dual luciferase assay established the interaction between AFAP1-AS1 and miR-508-3p. Furthermore, ZWINT, identified as a target of miR-508-3p, was significantly upregulated in lung adenocarcinoma tissues. Overexpression of ZWINT rescued the inhibitory effects of AFAP1-AS1 silencing on cell proliferation, colony formation, and apoptosis, while also reversing the reduction in cell invasion. AFAP1-AS1 accelerates the development of lung adenocarcinoma by cell proliferation, apoptosis, and invasion via the miR-508-3p/ZWINT axis. Thus, targeting AFAP1-AS1 or its downstream regulatory axis could offer novel therapeutic approaches in lung adenocarcinoma treatment.

## 1. Introduction

Lung cancer is the most common cause of cancer-related mortality globally, with an estimated 2.2 million new diagnoses and 1.8 million deaths annually. Although diagnosis and treatment have been improved, the prognosis of the lung cancer is still unfavorable, especially in the last stage [1]. Among its subtypes, lung adenocarcinoma has emerged as the most common histological form, accounting for over 40% of cases [2]. This variant, with glandular features and mucin metabolism, has exhibited a significant increase in relative prevalence, resulting from altered risk factors and refinement of histological classification [3]. The rising trend of lung adenocarcinoma draws to the surface the urgent need to further investigate its molecular mechanisms and discover new therapeutic targets.

Non-coding RNAs such as long non-coding RNAs (lncRNAs) and microRNAs (miRs) are attracting much attention as important mediators of gene expression involved in cancer. These molecules play vital roles in various cellular processes [4]. In particular, lncRNAs have been shown to play a role in oncogenesis via their function as competing endogenous RNAs (ceRNAs), as well as via miRNA sponging to modulate downstream gene expression [5,6]. Among such lncRNAs, AFAP1-AS1 has been reported as an oncogenic regulator in several cancers [7].

Recent findings indicate that AFAP1-AS1 exerts tumor-promoting action via some miRNAs’ interactions. For instance, AFAP1-AS1 controls the miR-195-5p/WISP1 axis to regulate colorectal cancer growth and metastasis [8]. In lung cancer, AFAP1-AS1 knockdown inhibits growth and promotes apoptosis [9]. Further, AFAP1-AS1 modulates the stemness and chemo-resistance of cervical cancer [10]. Another study suggests that inhibiting the expression AFAP1-AS1 suppresses tumorigenesis through apoptosis induction and suppression of the Wnt signaling pathway in NSCLC [11]. These results highlight the pleiotropic function of the AFAP1-AS1 in cancer development and AFAP1-AS1 as a therapeutic target.

However, the specific function of AFAP1-AS1 in lung adenocarcinoma is not clear enough. Based on this information, the current work explored the function of AFAP1-AS1 in the proliferation of human lung adenocarcinoma cells. In particular, we postulate that the AFAP1-AS1 regulates tumor progression through the miR-508/ZWINT axis. Through the elucidation of this pathway, we have the goal of providing new understanding of the molecular mechanisms underlying lung adenocarcinoma and discuss potential therapeutic approaches that address lncRNA–miRNA interactions.

## 2. Results

### 2.1. LncRNA AFAP1-AS1 Is Upregulated in Lung Adenocarcinoma

**Figure 1:**
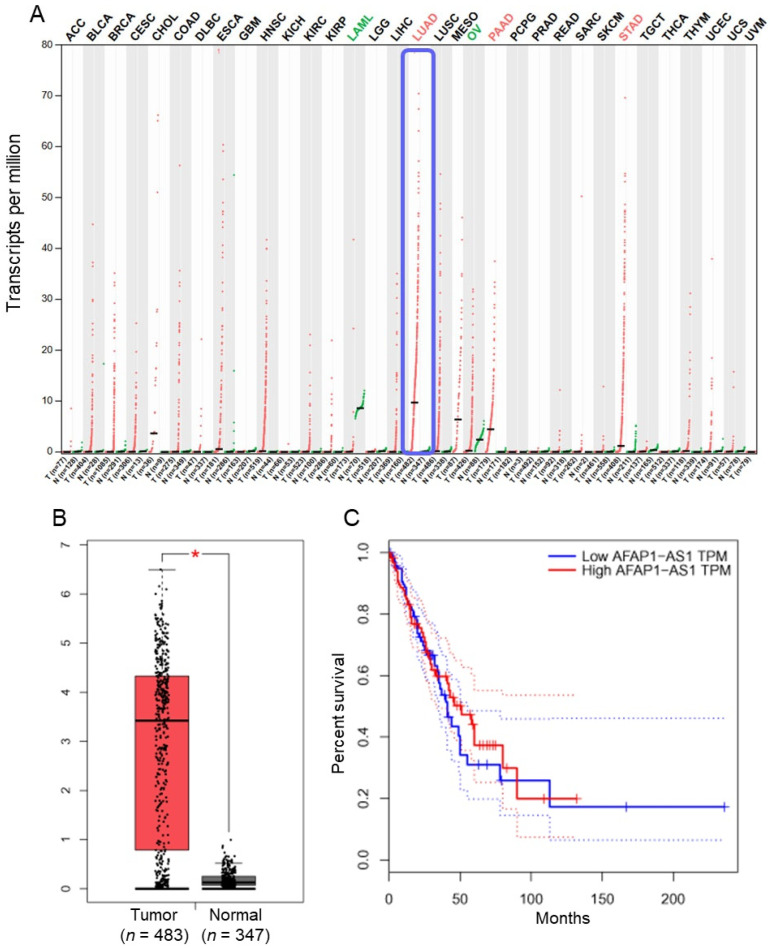
LncRNA-AFAP1-AS1 is upregulated in lung adenocarcinoma tissues: (**A**) expression profile of AFAP1-AS1 across various cancer tissues relative to normal tissues; (**B**) box plot illustrating expression levels of AFAP1-AS1 in lung adenocarcinoma (*n* = 483) versus normal tissues (n = 347); and (**C**) Kaplan–Meier survival curve comparing overall survival between low and high AFAP1-AS1 expression groups in lung adenocarcinoma patients (* *p* < 0.05).

**Figure 2:**
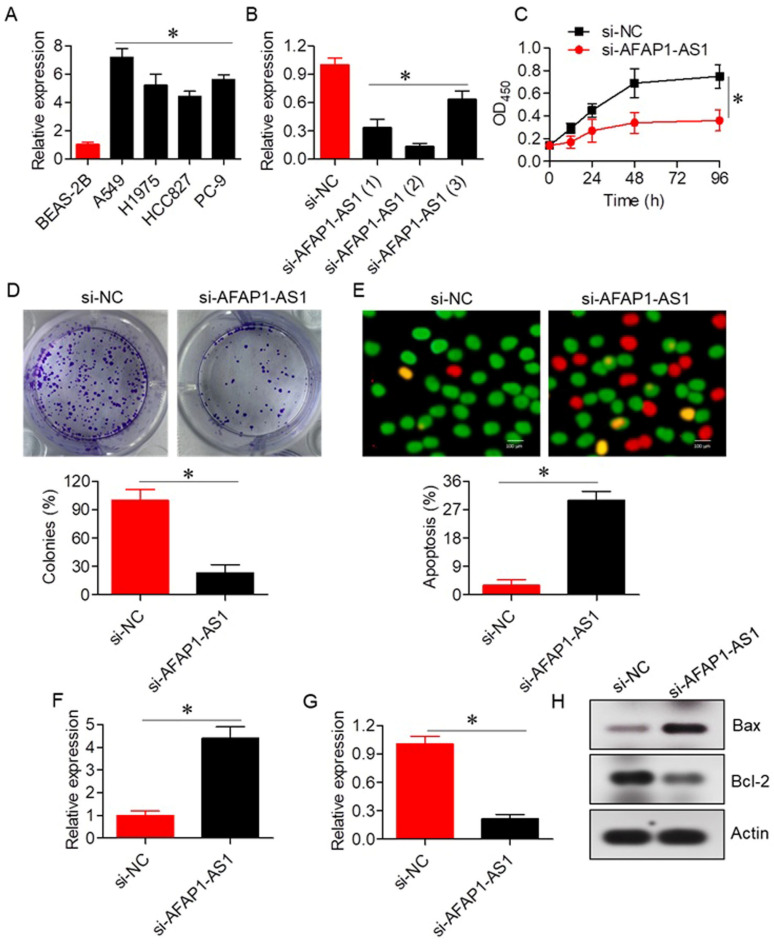
LncRNA-AFAP1-AS1 regulates proliferation of lung adenocarcinoma cells. (**A**) Expression of AFAP1-AS1 in normal lung and lung adenocarcinoma cell lines. (**B**) Expression of AFAP1-AS1 in A549 cells transfected with three different si-AFAP1-AS1 constructs. (**C**) Cell viability assay of A549 cells transfected with si-NC or si-AFAP1-AS1. (**D**) Colony formation assay of A549 cells transfected with si-NC or si-AFAP1-AS1. (**E**) AO/EB staining of A549 cells transfected with si-NC or si-AFAP1-AS1, highlighting apoptotic cells. (**F**) Expression of Bax in A549 cells transfected with si-NC or si-AFAP1-AS1. (**G**) Expression of Bcl-2 in A549 cells transfected with si-NC or si-AFAP1-AS1. (**H**) Western blots showing expression of Bax and Bcl-2 in A549 cells transfected with si-NC or si-AFAP1-AS1. Data are shown as mean ± SD (* *p* < 0.05).

The expression profile of AFAP1-AS1 across various human cancers was retrieved from the GEPIA database. It was observed that AFAP1-AS1 expression was significantly (*p* < 0.05) elevated in lung adenocarcinoma tissues compared to normal tissues (Figure 1A,B). However, Kaplan–Meier survival graphs comparing overall survival between low and high AFAP1-AS1 expression groups in lung adenocarcinoma patients showed no significant impact on overall survival (Figure 1C). Moreover, the expression of AFAP1-AS1 was analysed in normal BEAS-2B cells and lung adenocarcinoma cell lines (A549, H1975, HCC827, PC-9). The expression levels of AFAP1-AS1 were significantly (*p* < 0.05) higher in lung adenocarcinoma cells compared to normal BEAS-2B cells (Figure 2A).

### 2.2. Knockdown of LncRNA AFAP1-AS1 Impedes A549 Cell Proliferation

To elucidate the functional role of AFAP1-AS1, its expression was silenced using a siRNA-mediated approach with three distinct siRNA constructs (si-AFAP1-AS1 (1), si-AFAP1-AS1 (2), and si-AFAP1-AS1 (3)). While all three constructs effectively silenced AFAP1-AS1 expression, si-AFAP1-AS1 (2) induced the most significant reduction in AFAP1-AS1 expression (Figure 2B). Therefore, si-AFAP1-AS1 (2) was selected for subsequent experiments. The CCK-8 assay revealed that silencing AFAP1-AS1 resulted in a substantial decrease in A549 cell proliferation and colony formation (Figure 2C,D). The AO/EB assay confirmed that the anti-proliferative effects of si-AFAP1-AS1 were primarily due to increased apoptosis, with si-NC transfected A549 cells exhibiting around 3 ± 1.2% apoptosis, compared to 30 ± 4.4% apoptosis in si-AFAP1-AS1 transfected cells (Figure 2E). Additionally, silencing of AFAP1-AS1 led to enhanced Bax expression and decreased Bcl-2 mRNA and protein expression (Figure 2F–H).

### 2.3. LncRNA AFAP1-AS1 Acts via Sponging of miR-508-3p

**Figure 3:**
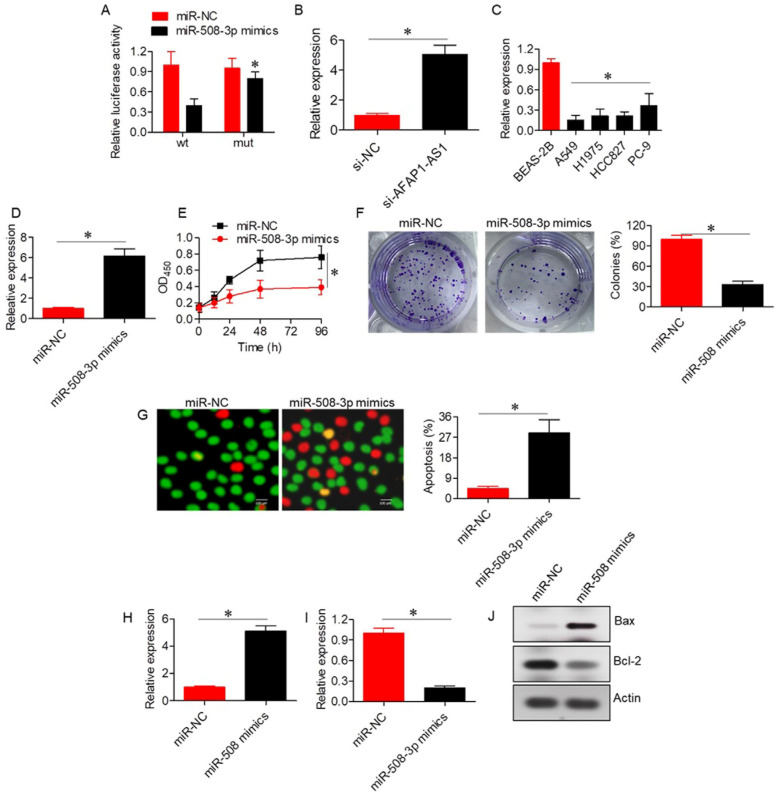
LncRNA-AFAP1-AS1 acts by sponging miR-508-3p. (**A**) Dual luciferase assay confirming the interaction between LncRNA-AFAP1-AS1 and miR-508-3p. (**B**) Expression of miR-508-3p in A549 cells transfected with si-NC or si-AFAP1-AS1. (**C**) Expression of miR-508-3p in normal lung and lung adenocarcinoma cell lines. (**D**) Expression of miR-508-3p in A549 cells transfected with miR-NC or miR-508-3p mimics. (**E**) Cell viability assay of A549 cells transfected with miR-NC or miR-508-3p mimics. (**F**) Colony formation assay of A549 cells transfected with miR-NC or miR-508-3p mimics. (**G**) AO/EB staining of A549 cells transfected with miR-NC or miR-508-3p mimics to assess apoptosis. (**H**) Expression of Bax in A549 cells transfected with miR-NC or miR-508-3p mimics. (**I**) Expression of Bcl-2 in A549 cells transfected with miR-NC or miR-508-3p mimics. (**J**) Western blots showing expression of Bax and Bcl-2 in A549 cells transfected with miR-NC or miR-508-3p mimics. Data are shown as mean ± SD (* *p* < 0.05).

The RNA22 V 2.0 online database predicted a potential interaction between AFAP1-AS1 and miR-508-3p (Supplementary Table S2), which was further validated via dual luciferase reporter assays (Figure 3A). Silencing of AFAP1-AS1 resulted in upregulation of miR-508-3p expression in A549 cells, confirming the interaction between AFAP1-AS1 and miR-508-3p (Figure 3B). Furthermore, the expression of miR-508-3p was significantly (*p* < 0.05) downregulated in lung adenocarcinoma cells, and its overexpression inhibited the proliferation and colony formation of A549 cells (Figure 3C–F). The inhibition of proliferation was associated with increased apoptosis, as evidenced by the AO/EB assay, and was accompanied by an increase in Bax and a decrease in Bcl-2 mRNA and protein expression levels (Figure 3G–J).

### 2.4. ZWINT Acts as a Target of miR-508-3p

**Figure 4:**
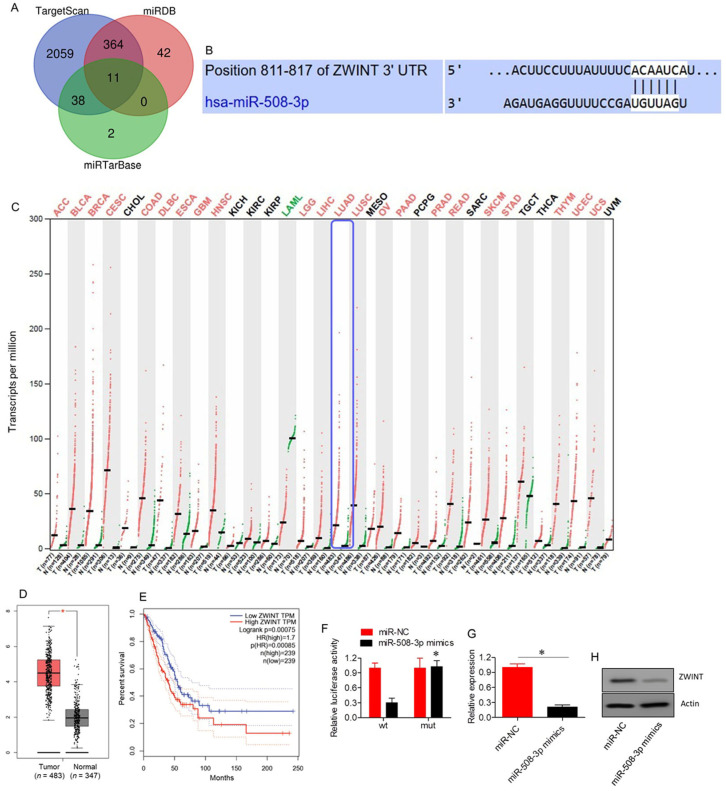
ZWINT targeted by miR-508-3p in lung adenocarcinoma. (**A**) Venn diagram illustrating common targets identified from three bioinformatics databases. (**B**) TargetScan analysis predicting the interaction between miR-508-3p and ZWINT 3′UTR. (**C**) Expression profile of ZWINT in different human cancers. (**D**) Box plot showing ZWINT expression levels in lung adenocarcinoma (*n* = 483) compared to normal tissues (*n* = 347). (**E**) Kaplan–Meier survival curve comparing overall survival between low and high ZWINT expression groups in lung adenocarcinoma patients. (**F**) Dual luciferase assay showing interaction between miR-508-3p mimics and ZWINT. (**G**) mRNA expression levels of ZWINT in A549 cells transfected with miR-NC or miR-508-3p mimics. (**H**) Western blots showing expression of ZWINT in A549 cells transfected with miR-NC or miR-508-3p mimics. Data are shown as mean ± SD (* *p* < 0.05).

TargetScan, miRDB, and miRTarBase were used to predict miR-508-3p targets, resulting in a total of 2942 potential targets (Supplementary Table S3). Among these, 11 targets were common across all three databases, as shown in the Venn diagram (Figure 4A; Supplementary Table S4). Notably, ZWINT was the only target that showed significant (*p* < 0.05) upregulation in lung adenocarcinoma tissues relative to normal tissues, as retrieved from the GEPIA database (Figure 4B–D). Furthermore, Kaplan–Meier survival assessments revealed that high ZWINT expression was associated with poor overall survival in lung adenocarcinoma patients (Figure 4E). ZWINT was subsequently identified as a target of miR-508-3p. The interaction between miR-508-3p and ZWINT was confirmed through dual luciferase reporter assays (Figure 4F). Inhibition of ZWINT expression in miR-508-3p mimics-transfected A549 cells further corroborated ZWINT as a target of miR-508-3p (Figure 4G,H).

### 2.5. ZWINT Overexpression Rescues the Effects of si-AFAP1-AS1

**Figure 5:**
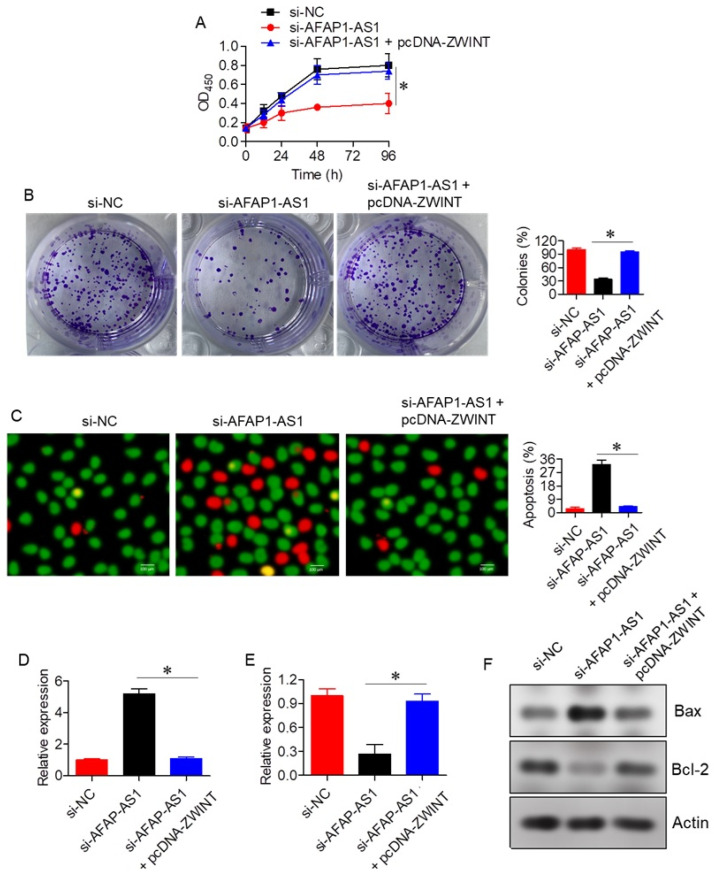
Effect of ZWINT on lung adenocarcinoma proliferation. (**A**) Cell viability of A549 cells transfected with si-NC, si-AFAP1-AS1, or si-AFAP1-AS1 + pcDNA-ZWINT. (**B**) Colony formation of A549 cells transfected with si-NC, si-AFAP1-AS1, or si-AFAP1-AS1 + pcDNA-ZWINT. (**C**) AO/EB staining of A549 cells transfected with si-NC, si-AFAP1-AS1, or si-AFAP1-AS1 + pcDNA-ZWINT, highlighting apoptotic cells. (**D**) Expression of Bax in A549 cells transfected with si-NC, si-AFAP1-AS1, or si-AFAP1-AS1 + pcDNA-ZWINT. (**E**) Expression of Bcl-2 in A549 cells transfected with si-NC, si-AFAP1-AS1, or si-AFAP1-AS1 + pcDNA-ZWINT. (**F**) Western blots showing expression of Bax and Bcl-2 in A549 cells transfected with si-NC, si-AFAP1-AS1, or si-AFAP1-AS1 + pcDNA-ZWINT. Data are shown as mean ± SD (* *p* < 0.05).

To assess whether AFAP1-AS1 regulates A549 cell proliferation through the miR-508-3p/ZWINT axis, the effects of ZWINT overexpression were evaluated in the context of AFAP1-AS1 silencing. Overexpression of ZWINT significantly (*p* < 0.05) reversed the growth inhibitory effects induced by AFAP1-AS1 silencing, as evidenced by results from proliferation, colony formation, and apoptosis assays (Figure 5A–C). Additionally, ZWINT overexpression restored the expression of Bax and Bcl-2 in si-AFAP1-AS1 transfected A549 cells (Figure 5D–F).

### 2.6. Effects of AFAP1-AS1 Silencing on A549 Cell Invasion

**Figure 6:**
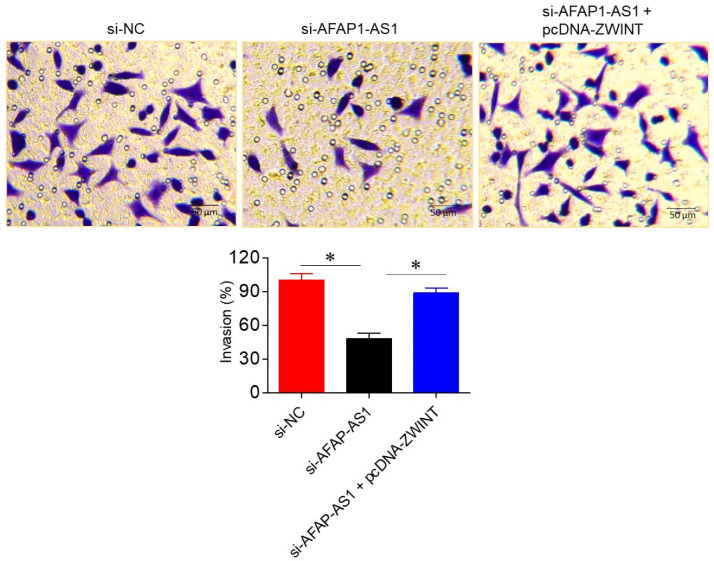
LncRNA-AFAP1-AS1 regulates invasion in lung adenocarcinoma Transwell assays, showing invasion of A549 cells transfected with si-NC, si-AFAP1-AS1, or si-AFAP1-AS1 + pcDNA-ZWINT. Data are shown as mean ± SD (* *p* < 0.05).

The influence of AFAP1-AS1 silencing on A549 cell invasion was assessed via Transwell assays. Silencing of AFAP1-AS1 significantly (*p* < 0.05) inhibited A549 cell invasion, with invasion levels in si-AFAP1-AS1 transfected cells being 48 ± 3.82% of the control si-NC transfected cells. Interestingly, overexpression of ZWINT was able to reverse the invasion inhibitory effects of AFAP1-AS1 silencing, further supporting the role of the miR-508-3p/ZWINT axis in regulating A549 cell invasion (Figure 6).

## 3. Discussion

Lung adenocarcinoma is one of the most prevalent and malignant lung cancers, having a dismal prognosis because of its early metastasis and drug resistance to standard therapies [12]. The biological processes of LncRNA have attracted considerable focus in recent years, such as the important role of cancer cell proliferation, apoptosis, and invasion [13]. LncRNAs possess the ability to regulate gene expression from multiple levels, and their dysregulation is often found in cancer [14]. Their promise as biomarkers and therapeutic targets opens new directions for cancer diagnosis and treatment [15]. This study aimed to investigate the role of AFAP1-AS1, an upregulated lncRNA in several cancers [7], and its functional mechanism in regulating apoptosis, cell proliferation, and invasion in lung adenocarcinoma cells.

In our results, AFAP1-AS1 was found to be markedly overexpressed in lung adenocarcinoma tissues and cells relative to normal tissues and cells. These results are in line with prior work, which reported the upregulation of AFAP1-AS1 in several cancers, such as pancreatic carcinoma [16], colon carcinoma, and lung carcinoma [17] and its correlation with adverse prognosis and malignant tumor traits. The Kaplan–Meier survival analysis in our study showed no significant effect of AFAP1-AS1 expression on overall survival in lung adenocarcinoma patients, although AFAP1-AS1 expression levels were correlated with tumor progression. This finding indicates that AFAP1-AS1 may play a greater role in the development and metastasis of the disease, similar to other cancers, such as nasopharyngeal carcinoma [18]. However, the lack of significant association between AFAP1-AS1 expression and overall survival may not serve as a robust independent prognostic biomarker.

Knockdown of AFAP1-AS1 using siRNA-mediated strategy significantly suppressed proliferation of A549 cells, both confirmed via CCK-8 and colony formation assays, thereby confirming that AFAP1-AS1 is involved in lung adenocarcinoma cell growth. This is consistent with previous reports in which AFAP1-AS1 silencing suppressed cell proliferation in several human cancers, such as colon cancers [19]. Additionally, we observed a marked increase in apoptosis following AFAP1-AS1 knockdown, as demonstrated by the AO/EB assay, with a substantial increase in apoptotic cells compared to controls. The apoptotic effects of AFAP1-AS1 silencing were further supported by an increase in Bax expression and a decrease in Bcl-2 expression, key markers of apoptosis regulation [20]. Bax is a pro-apoptotic protein inducing permeabilization of the mitochondrial outer membrane causing cytochrome c discharge and stimulation of downstream caspases, while Bcl-2 is an anti-apoptotic protein blocking Bax-induced apoptosis [21]. The alteration in the Bax/Bcl-2 ratio towards apoptosis as shown in this study suggests that AFAP1-AS1 can serve as a pro-survival factor by regulating pro-apoptotic signal transduction pathways, playing a role in the malignant phenotype of lung adenocarcinoma.

Apoptotic effects of AFAP1-AS1 silencing are probably attributed to the AFAP1-AS1 sponging function of miR-508-3p, which is supported by our dual-luciferase assay and RNA-expression analysis; miR-508-3p, previously shown to be downregulated in several cancers, including ovarian and gastric cancer [22,23], is a known modulator of apoptosis and cell proliferation. Our results indicate that AFAP1-AS1 upregulates the miR-508-3p expression through a mechanism that involves sequestering it from its target mRNA, and that might indirectly promote cell survival by decreasing apoptosis. All these observations have also been reported in the investigations of other lncRNAs. For example, LINC00511 functions as an apoptosis regulator in gastric cancer by binding with miRNAs [24]. Therefore, the miRNA-sponge activity of AFAP1-AS1 may explain its anti-apoptosis in lung adenocarcinoma.

Additional research discovered that miR-508-3p downregulates ZWINT, a subunit of the mitotic spindle checkpoint and division, previously shown to be associated with cancer progression [25]. ZWINT expression was also shown to be significantly upregulated in lung adenocarcinoma tissues; the relationship of ZWINT expression level with the prognosis was coincident with that of Peng et al. (2019) and Ma et al. (2023), who described the role of ZWINT in contributing to tumor growth by controlling cell proliferation, migration, and invasion [26,27]. In our experiment, overexpression of ZWINT reversed the inhibitory roles of AFAP1-AS1 knockdown on the proliferation and invasion of A549 cells, and this evidence suggests that AFAP1-AS1 may contribute to lung adenocarcinoma progression through the miR-508-3p/ZWINT axis. ZWINT has been associated with tumor metastasis, and upregulation has been demonstrated to support cell migration and invasion in multiple malignancies, such as cervical and prostate cancers [27,28].

To investigate AFAP1-AS1′s involvement in the invasion of lung adenocarcinoma cells further, we carried out Transwell assays. Our findings revealed that silencing of AFAP1-AS1 dramatically suppressed the invasive behaviour of A549 cells. This result is in agreement with previous studies in which AFAP1-AS1 knockdown alleviated invasion and metastasis in several cancers, such as nasopharyngeal carcinoma [18]. The invasive potential of lung adenocarcinoma cells was reversed in the presence of overexpression of ZWINT, thus demonstrating the applicability of intervention in limitation of tumor metastasis using the AFAP1-AS1/miR-508-3p/ZWINT axis.

While this study elucidates the downstream effects of AFAP1-AS1 through the miR-508-3p/ZWINT axis, the upstream regulatory mechanisms controlling AFAP1-AS1 expression remain largely unexplored. Previous studies have identified oncogenic transcription factors such as c-Myc, and SP1, as well as epigenetic modifiers like EZH2, as important regulators of lncRNA expression [29,30,31]. Notably, EGFR and KRAS mutations, which are common in lung adenocarcinoma, activate key signaling cascades such as MAPK/ERK and PI3K/AKT, which in turn modulate the expression of several oncogenic lncRNAs [32,33]. It is plausible that AFAP1-AS1 upregulation in lung adenocarcinoma may be driven by these signaling events, thereby integrating it into a broader oncogenic regulatory network that facilitates tumor progression. Future investigations into transcription factor binding at the AFAP1-AS1 promoter, or histone modification profiling, could clarify its upstream regulation and inform therapeutic strategies.

Though effective insights from this study can be drawn, there are certain limitations. First, our research primarily relied on the A549 cell line, and while this is a commonly used model for lung adenocarcinoma, it may not fully represent the molecular diversity of this cancer type. It is recommended that future investigations include a broader range of lung cancer cell lines and clinical tissue samples to represent the other subtypes of lung adenocarcinoma and to confirm the generalizability of our results. Moreover, although in vitro data show much promise, in vivo studies are required to validate the therapeutic effects of AFAP1-AS1 targeting or its downstream signaling as treatment for lung adenocarcinoma. Evenly, while we found a potential target (ZWINT) of miR-508-3p, more studies should be carried out to comprehensively understand the molecular roles of miR-508-3p and ZWINT in the progression of lung adenocarcinoma.

## 4. Materials and Methods

### 4.1. Cell Lines and Culture

Normal BEAS-2B cells and lung adenocarcinoma cell lines (A549, H1975, HCC827, PC-9) were obtained from ATCC. BEAS-2B cells were grown in bronchial epithelial medium (Lonza), while lung adenocarcinoma cell line cultures were grown in RPMI-1640 medium supplemented with 10% fetal bovine serum and 1% penicillin-streptomycin. Cells were cultured in standard conditions (37 °C, 5% CO_2_, 95% humidity).

### 4.2. Transfection with Constructs

Si-NC (negative control siRNA); si-AFAP1-AS1 (AFAP1-AS1 target); miR-NC (negative control miRNA); miR-508-3p mimics (miR-508 overexpression); and pcDNA-ZWINT (ZWINT overexpression) were used. Lipofectamine 3000 reagent was employed for transfection following the manufacturer’s guidelines. Transfection efficiency was allowed to reach 48 h prior to the examination of downstream experiments.

### 4.3. Quantitative Real-Time PCR (qRT-PCR)

Total RNA isolation was conducted using TRIzol reagent. DNA was synthesized using the PrimeScript™ RT Master Mix. QRT-PCR was carried out with SYBR^®^ Premix Ex Taq™ II on StepOnePlus^TM^ Real-Time PCR System. Relative expression of AFAP1-AS1, miR-508-3p, and ZWINT was standardized with GAPDH or U6. Data analysis was performed using the 2^ΔΔCt^ method. Primers are provided in Supplementary Table S1.

### 4.4. CCK8 Assay

Cell viability was evaluated using the CCK8 assay (Dojindo, Japan). A549 cells were plated in 96-well plates (5 × 10^3^ cells/well). At 24, 48, and 72 h, 10 µL of CCK8 solution was given together with transfection. After incubation at 37 °C for 2 h, absorbance at 450 nm was determined using a microplate reader.

### 4.5. Colony Formation Assay

A549 cells were grown at 1 × 10^3^ cells/well in 6-well plates and cultured for 2–3 weeks. After colonies were formed, fixing of cells was performed using 4% paraformaldehyde and staining with crystal violet solution. Colonies with more than 50 cells were counted using an inverted microscope.

### 4.6. Acridine Orange/Ethidium Bromide (AO/EB) Assay

Apoptosis of the cell was determined via AO/EB staining. A549 cells were plated and incubated in AO/EB (10 µg/mL) solution for 10 min. Cells were examined under a fluorescence microscope. Live cells, early apoptotic, and late apoptotic cells show green, yellow, and red fluorescence.

### 4.7. Transwell Assay

Invasion of A549 cells were quantitated using Transwell chambers. Two × 10^4^ cells were transfected and seeded in the upper chamber. RPMI-1640 medium with 10% FBS supplemented was used in the lower chamber. At 48 h of incubation, the cells which had invaded membranes were fixed with 4% paraformaldehyde and stained with crystal violet. Cells were examined and counted under a microscope.

### 4.8. Bioinformatics

From TargetScan, miRDB, and miRTarBase databases, ZW10 Interacting Kinetochore Protein (ZWINT) was identified as a candidate target of miR-508-3p. The expression levels of AFAP1-AS1 and ZWINT were also downloaded from the Gene Expression Profiling Interactive Analysis (GEPIA) database. AFAP1-AS1 and miR-508-3p interaction was assessed via the RNA22 V 2.0 database.

### 4.9. Dual Luciferase Assay

To confirm interactions of AFAP1-AS1 and miR-508-3p, as well as miR-508-3p and ZWINT, dual luciferase assays were carried out. AFAP1-AS1 and ZWINT 3′UTR regions, with the potential miR-508-3p binding sites predicted, were cloned into the pmirGLO Dual-Luciferase miRNA Target Expression Vector. A549 cells were co-subjected to transfection with luciferase reporter plasmids and miR-508-3p mimics or miR-NC. Luciferase activity was determined at 48 h using the Dual-Luciferase Reporter Assay System. Firefly luciferase activity was used to normalize Renilla luciferase activity.

### 4.10. Western Blotting

Protein expression was assessed through western blotting. Firstly, lysing of the cells was performed using RIPA buffer, and subsequently BCA assay was conducted to measure the protein concentrations. Following this, loading of equal protein quantities onto an SDS-PAGE gel was carried out. This was followed by transference to a PVDF membrane, and blocking with 5% milk in TBS-T. The membrane was incubated with a specific primary antibody (ZWINT, Cat #MA5-17284, β-actin, Cat #MA5-15739, ThemoFisher Scientific, Waltham, US) overnight at 4 °C, and then with a HRP-conjugated secondary antibody for 1 h. Finally, washing was performed and protein bands were detected using an ECL substrate and captured with a chemiluminescence imaging system. The intensity of the bands was then normalized to β-actin.

### 4.11. Statistical Analysis

Statistical analysis was conducted using GraphPad Prism (version 9.5.1) software. Results are shown as mean ± standard deviation (SD) from at least three independent biological replicates. Prior to applying statistical tests, data were assessed for normality using the Shapiro–Wilk test. For group comparisons, Student’s *t*-test or one-way ANOVA followed by Tukey’s post-hoc test was used, and *p* < 0.05 was taken as statistically significant.

## 5. Conclusions

In conclusion, our study revealed that AFAP1-AS1 plays a critical role in promoting the progression of lung adenocarcinoma through its interaction with miR-508-3p and subsequent regulation of ZWINT. The AFAP1-AS1/miR-508-3p/ZWINT axis contributes to key oncogenic processes including cell proliferation, apoptosis suppression, and invasion, underscoring its potential as a novel therapeutic target in lung cancer. These findings highlight the importance of further exploring the molecular mechanisms underlying lncRNA-mediated regulation in cancer and supporting the ongoing development of lncRNA-based therapeutic strategies.

## Data Availability

The raw data supporting the conclusions of this article will be made available by the authors on request.

## Supplementary Materials

The following supporting information can be downloaded at https://www.mdpi.com/article/10.3390/ijms26136532/s1.
