# Supplementary material for: Regulation of Human Lung Adenocarcinoma Cell Proliferation by LncRNA AFAP-AS1 Through the miR-508/ZWINT Axis
Source: Int J Mol Sci. 2025 Jul 7;26(13):6532. doi: 10.3390/ijms26136532 (PMC12249709; doi:10.3390/ijms26136532)
Supplement: Supplementary file 1 [file ijms-26-06532-s001.zip › ijms-3717313-supplementary.pdf]

Supplementary Table 1: List of primers used in the study.

Primer	Direction	Sequence
miR-508-3p	Forward	ACTGTATGATTGTAGCCTTTTGG
	Reverse	TATGGTTTTGACGACTGTGTGAT
U6	Forward	CTCGCTTCGGCAGCACA
	Reverse	AACGCTTCACGAATTTGCGT
AFAP1-AS1	Forward	GGGCUUCAAUUUACAAGCATT
	Reverse	UGCUUGUAAAUUGAAGCCCTT
GADPH	Forward	CGTAAAGACCTCTATGCCAACA
	Reverse	TAGGAGCCAGGGCAGTAATC
Bax	Forward	TCAGGATGCGTCCACCAAGAAG
	Reverse	TGTGTCCACGGCGGCAATCATC
Bcl-2	Forward	ATCGCCCTGTGGATGACTGAGT
	Reverse	GCCAGGAGAAATCAAACAGAGGC

Supplementary Table 2: RNA22 Version 2.0 (http://cm.jefferson.edu/rna22/Precomputed/) confirming interaction of LncRNA-AFAP1-AS1 with miR-508-3p.

miRNA identifier	Left most position of predicted target site	Folding energy (Kcal/mol)	Predicted target site	Targeting miRNA	Base pairing-LncRNA	Base pairing-miRNA	Base pairs in putative heteroduplex	**P-value**
hsa_miR_508_3p	1322	-14.50	ACATTTCTTTAAGGCACATTCA	TGATTGTAGCCTTTTGGAGTAGA	....((((..((((((((.(((	))).))).)))))..))))....	15	0.246000
hsa_miR_508_3p	4165	-16.50	CTTGCTCCATGACTCTGCAAGCA	TGATTGTAGCCTTTTGGAGTAGA	.((((((((.((..((((((.((	)).))))))..)).)))))))).	18	0.239000
hsa_miR_508_3p	4589	-12.10	CCTGAGGAGAGGCGAAGCATTCG	TGATTGTAGCCTTTTGGAGTAGA	.(((..(((((((...(((.(((	))).))).))))))))....))).	16	0.081200
hsa_miR_508_3p	6420	-13.12	TCTGGGCTTCAATTTACAAGCAGTCA	TGATTGTAGCCTTTTGGAGTAGA	(((..(((((((.......((((((	))))))).......))))))))))	17	0.109000

Supplementary Table 3: Total and common targets of miR-508-3p among three Databases

List names	Number of targets
TargetScan	2474
miRDB	417
miRTarBase	51
Common targets	11

Supplementary Table 4: Identification of common targets of miR-miR-508-3p identified from three different online databases (TargetScan, miRDB, and miRTarBase)

Target gene	Representative transcript	Gene name
**ZBTB18**	ENST00000358704.4	zinc finger and BTB domain containing 18
**MOB4**	ENST00000233892.4	MOB family member 4, phocein
**ZWINT**	ENST00000373944.3	ZW10 interacting kinetochore protein
**SOCS3**	ENST00000330871.2	suppressor of cytokine signaling 3
**CEP19**	ENST00000409690.3	centrosomal protein 19kDa
**NFKB1**	ENST00000226574.4	nuclear factor of kappa light polypeptide gene enhancer in B-cells 1
**PCMT1**	ENST00000367384.2	protein-L-isoaspartate (D-aspartate) O-methyltransferase
**FLOT2**	ENST00000394906.2	flotillin 2
**HMGA2**	ENST00000403681.2	high mobility group AT-hook 2
**MBNL1**	ENST00000357472.3	muscleblind-like splicing regulator 1
**SRSF1**	ENST00000258962.4	serine/arginine-rich splicing factor 1
